# Impacts of Different Exposure Scenarios on Transcript Abundances in *Danio rerio* Embryos when Investigating the Toxicological Burden of Riverine Sediments

**DOI:** 10.1371/journal.pone.0106523

**Published:** 2014-09-04

**Authors:** Kerstin Bluhm, Jens C. Otte, Lixin Yang, Christian Zinsmeister, Jessica Legradi, Steffen Keiter, Thomas Kosmehl, Thomas Braunbeck, Uwe Strähle, Henner Hollert

**Affiliations:** 1 Department of Ecosystem Analysis, Institute for Environmental Research, Aachen Biology and Biotechnology, RWTH Aachen University, Aachen, Germany; 2 Institute of Toxicology and Genetics, Karlsruhe Institute of Technology, Karlsruhe, Germany; 3 Institute for Environmental Studies, VU University Amsterdam, Amsterdam, The Netherlands; 4 Aquatic Ecology and Toxicology Group, Center for Organismal Studies, University of Heidelberg, Heidelberg, Germany; 5 School of Environment, Nanjing University, Nanjing, China; 6 Key Laboratory of Yangtze River Environment of Education Ministry of China, College of Environmental Science and Engineering, Tongji University, Shanghai, China; 7 College of Resources and Environmental Science, Chongqing University, Chongqing, China; National University of Singapore, Singapore

## Abstract

**Purpose:**

Recently, a proof-of-concept study revealed the suitability of transcriptome analyses to obtain and assess changes in the abundance of transcripts in zebrafish (*Danio rerio*) embryos after exposure to organic sediment extracts. The present study investigated changes in the transcript abundance in zebrafish embryos exposed to whole sediment samples and corresponding organic extracts in order to identify the impact of different exposure pathways on sediment toxicity.

**Materials and Methods:**

*Danio rerio* embryos were exposed to sublethal concentrations of three sediment samples from the Danube River, Germany. The sediment samples were investigated both as freeze-dried samples and as organic extracts. Silica dust and a process control of the extraction procedure were used as references. After exposure, mRNA was isolated and changes in profiles of gene expression levels were examined by an oligonucleotide microarray. The microarray results were compared with bioassays, chemical analysis of the sediments and profiles of gene expression levels induced by several single substances.

**Results and Discussion:**

The microarray approach elucidated significant changes in the abundance of transcripts in exposed zebrafish embryos compared to the references. Generally, results could be related to Ah-receptor-mediated effects as confirmed by bioassays and chemical analysis of dioxin-like contaminants, as well as to exposure to stress-inducing compounds. Furthermore, the results indicated that mixtures of chemicals, as present in sediment and extract samples, result in complex changes of gene expression level profiles difficult to compare with profiles induced by single chemical substances. Specifically, patterns of transcript abundances were less influenced by the chemical composition at the sampling site compared t the method of exposure (sediment/extract). This effect might be related to different bioavailability of chemicals.

**Conclusions:**

The apparent difference between the exposure scenarios is an important aspect that needs to be addressed when conducting analyses of alterations in the expression level of mRNA.

## Introduction

Genomic technologies have repeatedly been used in studies investigating ecotoxicological impacts on aquatic organisms as reviewed by Piña & Barata [1]. In contrast to studies into individual contaminants, few studies have been conducted for defined chemical cocktails (e.g., for resin acids [2], metals [3], PAHs [4] or complex mixtures of chemical compounds as found in effluents [5], river sediments [6] and river estuaries [7], [8]. Very recently Kosmehl et al. [9] could document gene expression level profiling of zebrafish embryos as a useful tool for the investigation of complex contaminated environmental samples such as sediment extracts. Their results indicated that contaminant classes might be assignable to sediment extracts by the use of classical biomarker genes and by correlating profiles of expression levels of single substances that were previously reported. Nevertheless, only few alterations in the abundance of transcripts could be explained by analytical chemistry or biological effects.

The fish embryo toxicity test with zebrafish (*Danio rerio*) is a common ecotoxicological biotest that is used to determine embryo toxicity and teratogenicity of chemicals and water samples but provides little information about the mechanisms of toxicity. It was further developed as a sediment contact assay for the assessment of sediment toxicity [10] as well as the genotoxic potential of sediments [11]. In this context, ecotoxicogenomic tools may provide a better mechanistic understanding of the impact of a chemical substance [12]. For example, gene expression studies provide an opportunity to identify new molecular biomarkers, but also to elucidate mechanisms of action of environmental contaminants [1], [13]. However, it is also necessary to face the problem of properly interpreting microarray data regarding variations in the test system such as whole sediment exposure *versus* exposure to sediment extracts. Whereas vigorous extractions simulate a ‘worst-case scenario’, biomimetic extractions or whole sediment exposure (also known as sediment contact assays) can yield insight into bioavailability of sediment contaminants [14]. In this context, the zebrafish embryo toxicity test was applied in the course of an effect-directed analysis of sediment extracts [15] and in combination with various extraction methods and sediment contact exposure to characterize the extraction method regarding their stringency and predictability for bioaccessibility [16]. Furthermore, regarding a differentiation between the bioavailable and the extractable fraction, Kosmehl et al. [17] introduced a test strategy exemplarily for the assessment of genotoxicity in the comet assay with zebrafish embryos: They concluded that there is a striking advantage in assessing the genotoxicity by means of different exposure scenarios, which focus on either bioavailable or extractable fractions, since the combination of the results provides information both on their bioavailability and specific properties of the genotoxicants.

This study was conducted within the framework of the DanTox project [18] with the aim (1) to identify the impact of different exposure pathways (freeze-dried sediments *versus* acetonic sediment extracts) on the abundance of transcripts and (2) to elucidate if changes in the abundance of transcripts of both exposure pathways can be linked to chemical analyses as well as results of bioassays performed with the same samples within a weight-of-evidence study conducted at the upper Danube River. This weight-of-evidence study had been designed to find an explanation for the local fish decline by combining investigations into cytotoxicity, dioxin-like activity, mutagenesis and genotoxicity of sediment samples [19], [20], [21], [22].

## Materials and Methods

### Samples and sample processing

**Figure 1:**
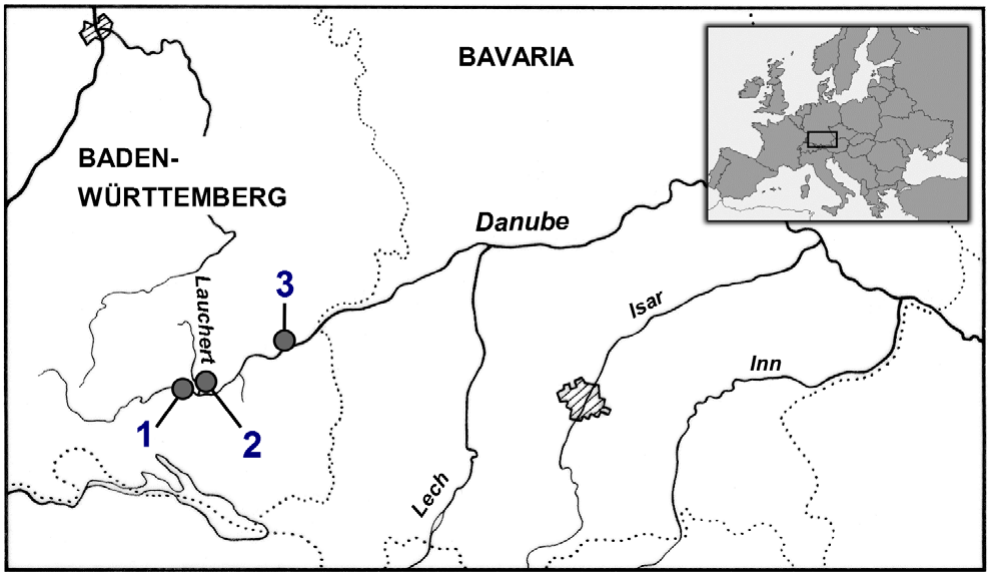
Sampling sites along the Danube river. 1 Sigmaringen, 2 Lauchert and 3 Ehingen. (Freely modified from [19], [20], [21], [53]).

The sediment samples were collected from two different sites (Sigmaringen and Ehingen) and one tributary (Lauchert) along the Danube River in Germany (Figure 1). Sampling and transport conditions as well as lyophilization of the samples were described in detail by Keiter et al. [20]. Based on results from a weight of evidence study, the three sediment samples were chosen for investigation in this study due to their different ecotoxicological potentials. With respect to a fuzzy logic approach to classify sediments on the basis of results from in vitro tests, the Lauchert sample was classified as a sediment with no to low potential risk, the Sigmaringen sample as a sediment with a critical ecotoxicological risk and the Ehingen sample as a sediment with a high to very high ecotoxicological risk [23]. The sediments were examined by microarray experiments both as freeze-dried samples (in the following referred to as whole sediments) and as organic extracts of the freeze dried samples. Organic extracts were prepared using a Soxhlet apparatus (for further details, see Hollert et al. [24]). For each sampling site, 20 g of the dried sediment samples were separately extracted with acetone (Fluka, Switzerland) for 16 h at approximately ten cycles per hour. The extraction was repeated with a second subsample of the sediment from Lauchert and Sigmaringen and the extracts were reduced close to dryness. The resulting extract concentration was adjusted to 20 g sediment equivalents dry weight per ml DMSO (Serva, Heidelberg, Germany) and extracts of the same sediment sample were combined to one. Extract concentrations used for the microarray experiments are given in mg sediment equivalent per ml (SEQ/ml). Silica dust (Millisil W4, Quarzwerke, Frechen, Germany) was used as a negative control and to dilute the whole sediment samples to a defined test concentration.

### *Danio rerio* embryo exposure to whole sediments and sediment extracts

Zebrafish were kept and bread as described by Westerfield [25]. For microarray analysis, the zebrafish strains AB, ABO, and Tübingen were combined and used.

Exposure was carried out analogously to the protocol of the fish embryo toxicity test [26] in the variation of the whole sediment protocol by Hollert et al. [10]. In order to allow for sufficient amounts of total RNA (minimum 200 ng/µl) and to reduce the individual variability, 200–600 embryos per sample concentration were exposed and pooled. For exposure 200 embryo were placed in individual 150×25 mm glass petri dishes (Schott, Mainz, Germany). If compared to Hollert et al. [10] and Strmac et al. [27], the number of embryos was five-fold higher in relation to the portion of sediment and dilution volume of the extracts, respectively. Embryo medium (60 µg/ml Instant Ocean, Red Sea, Houston, TX; pH 6.73, Ca 0.8 mg/l; K 0.6 mg/l; Mg 2 mg/l; Na 16 mg/l; S 2 mg/l) served as a negative control. Additional controls were silica dust and an extraction control (process control). The process control corresponded to the highest DMSO concentration applied for investigations of sediment extracts (0.15°% DMSO) Plates were sealed with a plastic paraffin film (Parafilm, American National Can, Chicago, IL, USA) to prevent evaporation. Each sample and control was exposed and tested three times; the Lauchert whole sediment was tested four times, silica dust was tested only twice. Exposure lasted from 4 to 96 hours post fertilization (hpf) at 27°C. The freeze-dried sediment and extract concentrations were adjusted to keep embryo mortalities below 20%. Range-finding tests revealed a test concentration for microarray analyses of 37.5 mg/ml for Ehingen whole sediment and of 300 mg/ml for Lauchert and Sigmaringen whole sediments. The extracts were tested at a concentration of 30 mg SEQ/ml for Lauchert and 10 mg SEQ/ml for Sigmaringen and Ehingen with a solvent concentration of 0.15% and 0.05%, respectively. Embryos were examined daily using a Nikon SMZ 645 microscope. Mortality and development were documented, and coagulated embryos were removed. At the end of each test, the pH was controlled for each petri dish.

### Purification of mRNA from zebrafish embryos

Total RNA was isolated from pooled zebrafish embryos (2 petri dishes) per treatment using the Machery-Nagel (MN, Düren, Germany) NucleoSpin RNA L isolation kits. After shock-freezing with liquid nitrogen, the tissue was disrupted using a ceramic pestle and mortar. The cell homogenate was transferred to 3.6 ml lysis buffer and the following steps were done according to the NucleoSpin RNA L isolation kit instructions. The final product yielded 260 nm/280 nm ratios of 1.9–2.1, and concentrations were determined based on absorbance at 260 nm. The integrity of total RNA was confirmed by denaturing agarose gel electrophoresis according to the RNeasy Mini Handbook (Qiagen, Hilden, Germany). mRNA was purified from total RNA samples and precipitated over night according to the Ambion MicroPoly(A) Purist™ Small Scale mRNA Purification Kit instructions (Huntingdon, UK).

### Microarray analysis

For labelling of the probes, cDNA was synthesised from 1.5–2.0 µg mRNA using the Amersham Cyscribe First-Strand cDNA Kit (Austin, TX, USA). The purification of labelled probes was performed according to the Microcon protocol (Millipore, Bedford, MA, USA). The quantity of labelled probe was estimated using a Nanodrop UV-Vis spectrophotometer (Wilmington, USA).

Microarray printing was done according to Yang et al. [3] and Kosmehl et al. [9]. The experimental design is based on a two-color gene expression assay. To avoid gene-specific dye effects, the experiment was replicated with reverse-labeling to balance the green and red dyes. The two microarrays with reverse-labeled probes were technical replicates.

Hybridization on the microarray chips, the following washing steps, scanning of the arrays, quality control during image acquisition, data pre-processing, transformation and normalisation were in accordance with procedures described by Yang et al. [3]. To identify differential expression levels in comparison to the untreated group, each individual gene was tested for a difference in expression level under exposure conditions with an adjusted t-test. Significant changes in the expression level were determined by a *p*-value smaller than 0.025. *P*-values were adjusted for multiple testing to avoid too many false positive genes. In order to account for this, a false discovery rate (FDR)-based Benjamin-Hochberg adjustment was used [28], since this reduces the number of false positives without being too conservative and, thereby, inflating the number of false negatives. Differences in transcript abundances for each treatment were displayed only, when there was an alteration by a factor of at least 2.0 fold-change (fc) at a significance level of *α* = 0.025. Fold-change values below 1 were replaced by the negative of its inverse.

Microarray annotation and GO-analyses were performed according to Kosmehl et al [9].

All microarray data from this study have been deposited in NCBI’s Gene Expression Omnibus under the accession number GSE31400.

### Clustering

Data of the three whole sediments, sediment extracts, silica dust and the process control was analysed and additionally compared to the patterns of eleven substances of a previous study [3] as well as to patterns of two sediment extracts from the river Rhine [9]. The data of the previous studies could easily been integrated in this study as processing and analysis was done using the same methodologies [3]. The cluster analysis was carried out using MATLAB R2010a (MathWorks, Natick, MA, USA) after loading the calculated logarithmic fold-change values into MATLAB. The gene selection for multivariate analysis was based on statistical parameters like adjusted *p*-values, fold-changes, median absolute deviation from the median (MAD) and Pearson correlations. A hierarchical agglomerative clustering algorithm was used. The clustering was based on changes in the expression of mRNA at a significance level of *α* = 0.05 and belonged to at least one of the following groups: the top 20 transcripts with significant changes in the abundance based on fold-change (minimum |fc| ≥2); top 100 transcripts with the highest MAD across all treatments and the abundance of transcripts of marker genes that are altered at least 3-fold for only one treatment. Differences in the abundance of transcripts for particular treatments are illustrated by a colour map ranging from −5 to +5. Blue segments represent genes with a strong decrease in the abundance of the corresponding transcripts, whereas red stands for genes with a strong increase in the abundance of the corresponding transcripts. Green segments denote unaffected genes.

### Ethics statement

All experiments were conducted in accordance with the Animal Welfare Act and with permission of the federal authorities (Regierungspräsidium Karlsruhe, Germany and Landesamt für Natur, Umwelt und Verbraucherschutz NRW, Germany). Moreover, according to the EU Directive 2010/63/EU on the protection of animals used for scientific purposes, early life-stages of zebrafish are not protected as animals until the stage of being capable of independent feeding (5 days post fertilization). In this study the experiments did not exceed an exposure time of 4 days post fertilization, thus, the zebrafish utilized were not capable of independent feeding and not protected as animals according to the EU Directive mentioned above.

## Results

### Embryo toxicity and teratogenicity

Both exposure scenarios resulted in mortality rates <20%. However, sublethal effects were observed: Spinal deformations were detected in all treatments as well as in negative control groups, but in less than 20% of the embryos. For exposure to whole sediments and the associated negative controls, no differences were found between the number of spinal deformations (less than 10%). For exposure to sediment extracts, a higher number of embryos showed spinal deformations (less than 20%) compared to the negative control (less than 10%). Further obvious morphological effects above a rate of 5% were not observed for the concentrations used.

### Transcript abundance of genes after exposure to whole sediments and sediment extracts

Genes corresponding to transcripts with significant changes in the abundance (2-fold change; adjusted *p*-value: 0.025) are listed in Table S1. With the exception of the silica dust treatment, all treatment groups revealed changes in the abundance of transcripts compared to the control. As a reference for extract treatments, the process control induced only significant alterations in the abundance of transcripts of *ela2l*. In comparison to this treatment reference, significant alterations in the abundance of transcripts were calculated to a greater extent for the sediment extract treatments. Among them, sediment extracts from Lauchert and Sigmaringen revealed changes in transcript abundance for a higher number of genes (30 and 21 genes, respectively) in comparison to the whole sediments from the same sample sites (24 and 13 genes, respectively). Only for the sample from Ehingen, changes in transcript abundance were revealed for a lower number of genes in the extract treatment compared to the whole sediment treatment (6 and 16, respectively).

The number of transcripts with increased abundance exceeded the number of transcripts with decreased abundance when testing whole sediments (Lauchert: 22 compared to 2; Sigmaringen: 11 compared to 2; Ehingen: 12 compared to 4). With regard to the sediment extracts, only the Ehingen extract induced a higher number of transcripts with increased abundance than decreased abundance (5 compared to 1). For the remaining extract treatments and the process control, respectively, more transcripts with decreased abundance than increased abundance were found.

Among the transcripts that showed altered abundance in all whole sediment treatments the absolute-value of fold-changes was approximately equal with the exception of the fold-changes for *annexin A1b* (*anxa1b*). The abundance of this transcript was stronger altered after treatment with Ehingen whole sediment (fc: −5.3 compared to fc: −4.3 and fc: −2.6 for the Lauchert and Sigmaringen whole sediment samples, respectively, Table S1). With regard to the sediment extract treatments, changes in transcript abundances were stronger compared to the whole sediment exposures and predominantly stronger after treatment with Lauchert or Sigmaringen extract compared to the treatment with Ehingen extract.

A comparison of significant alterations in the abundance of transcripts (fc≥2.0; adjusted p-value≤0.025) found after whole sediment treatments with significant alterations in the abundance of transcripts after treatments with the corresponding extracts revealed a comparatively low consistency. In contrast, higher similarity of alterations in the abundance of transcripts was found among the whole sediment treatments as well as among the extract treatments. By taking into account significant alterations in the expression level of mRNA for the three whole sediment treatments similarities in alteration could be found for 17.6% of the transcripts. For the comparison of all extract treatments the percentage of matching direction of changes in transcript abundance was 15.6%. The comparison of only two whole sediments or two sediment extracts revealed even higher degrees of similarities in the abundance of transcripts, e.g., 45% for Sigmaringen and Ehingen whole sediment and 59% for Lauchert and Sigmaringen sediment extract. These are all higher percentages compared to the results of whole sediments and sediment extracts from the same sampling site. For the whole sediment and the sediment extract of the tributary Lauchert, only 3.8% of the significant changes in transcript abundance were found in both exposure scenarios. Whole sediment and sediment extract from Sigmaringen revealed 9.7% and from Ehingen 10% similarity in transcript abundance. Thus, the total number of genes showing significant changes in the abundance of transcripts was several times lower than found for the comparison of the whole sediments and sediment extracts of different sample sites.

**Figure 2:**
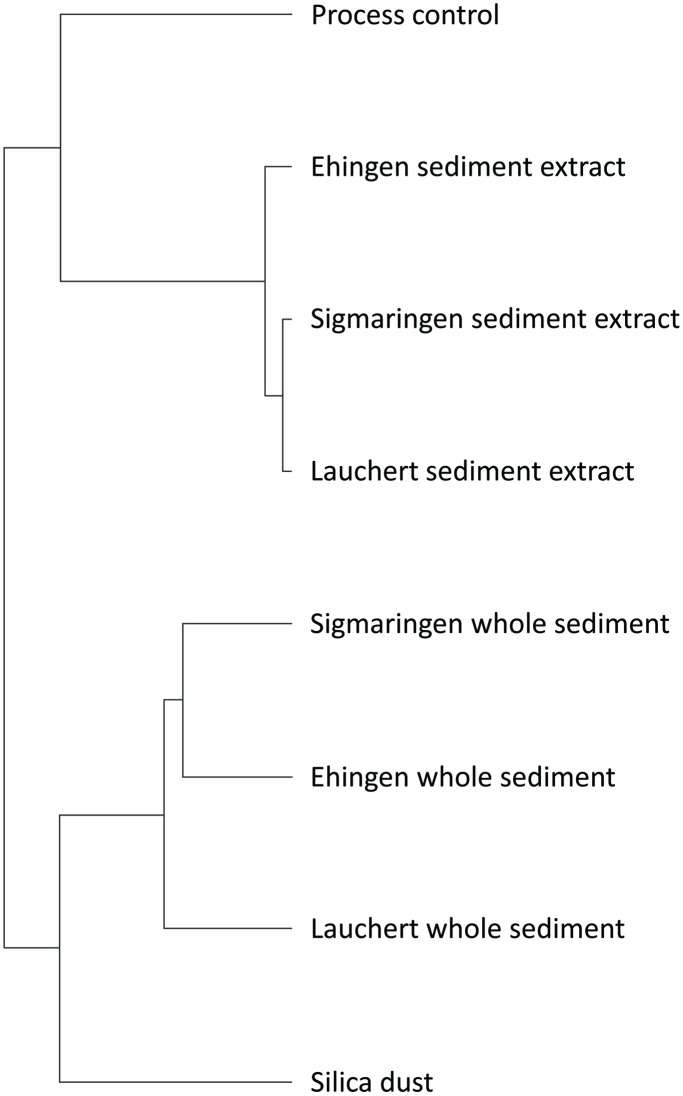
Dendrogram obtained by cluster anaylsis of whole sediment and sediment extract treatments as well as of silica dust and the process control treatment.

If compared to the evaluation of changes in transcript abundance of individual genes, cluster analysis illustrated the same results. Two main clusters were identified (Figure 2): The first cluster contained all whole sediment treatments as well as silica dust, whereas in the second cluster all extract treatments as well as the process control were located (Figure 2). Among the whole sediments, Sigmaringen and Ehingen showed the strongest similarity to each other compared to the Lauchert sediment. The extracts are differently clustered, with the Lauchert and Sigmaringen extracts indicating a stronger similarity to each other than to the Ehingen extract. The references silica dust and process control showed very low similarity among one other. Furthermore, they showed a lower degree of similarity to the whole sediments and extracts, respectively, compared to the whole sediment or sediment extract samples among one another.

### Selected genes with changes in the abundance of their transcript after whole sediment and sediment extract treatments

As shown in Table S1, some genes with changes in the abundance of their transcript after treatment with whole sediments or sediment extracts belong to a set of genes encoding proteins with similar functions. Remarkable is a set of genes encoding information on digestive enzymes (Table S1, A: green background) and a set of genes encoding phase I and phase II enzymes that are involved in detoxification of xenobiotics (Table S1, B: purple background). The transcript abundance of the genes encoding such proteins was increased. Among them was a gene with the strongest increase in the level of mRNA (fold-changes between 3.0 and 44.0) in this study. This gene encodes the phase I enzyme cytochrome P450 1A (CYP1A; Table S1) and the corresponding transcript level was altered for all samples but the fold-change was lower for whole sediments than for extracts. Among the whole sediments, there was a similar regulation of *cyp1a* for Sigmaringen and Ehingen (fold-change of 5.1 and 5.2) and a lower regulation for Lauchert (fold-change of 3.0). In contrast, the abundance of transcripts of *cyp1a* was similar following treatment with Lauchert extract and Sigmaringen extract (fold-changes of 43.3 and 44, respectively). For the treatment with Ehingen sediment extract a lower alteration could be found (fold-change of 21.2). The transcript abundance of the gene *gst* was only altered for extract exposures with fold-changes ranging between 2.2 and 2.4. This gene encodes the phase II enzyme glutathione S-transferase pi. In contrast to the increased abundance of transcripts encoding phase I and II enzymes, the transcript abundance of nearly all genes encoding information on digestive enzymes or enzymes involved in lipid catabolism was decreased (Table S1, gene set A). The abundance of transcripts of these genes was mainly altered after extract treatments. However, the Ehingen extract induced changes in the abundance of transcripts only for some of these genes and solely considering fold-changes between 2.0 and 1.7 in combination with an adjusted *p*-value<0.005. For one of the corresponding genes, *ela2l*, differences in transcript abundance were significantly decreased (fold-change of −2.2) in the process control. Taking again into account fold-changes between 2.0 and 1.7 with an adjusted *p*-value<0.005, transcripts of two further genes of the gene set Table S1, A were decreased for the process control.

## Discussion

Microarray approaches should show results also at low test concentrations. To verify this and to assure that differences in the abundance of transcripts are not compromised by acute toxicity, concentrations below the EC_20_ values were chosen for this study. As a result, the overall number of transcripts with a significant alteration in abundance was low compared to other studies by Yang et al. [3] or Kosmehl et al. [9] due to the relatively low sediment concentrations tested in this study. Silica dust controls caused no significant changes in the abundance of transcripts at our settings. This implies that silica dust is an appropriate material for the dilution of whole sediments to be tested in cDNA-microarray experiments, as it will not specifically alter the abundance of transcripts. In contrast to the other samples (n = 3; n = 4 for Lauchert whole sediment), only two replicates were available for analysis of silica dust, and therefore, a statistical analysis is questionable. However, a manual comparison of the fold-changes of genes with significant alterations in the expression level of mRNA after whole sediment treatments with the fold-changes obtained for these genes after exposure with silica dust revealed lower alterations for silica dust. The fold-changes after treatment with silica dust mainly ranged between −1.6 and 1.6. Only the abundance of transcripts of three genes exceeded these values. These are genes with unknown identity (AI353541, AI437134 and BE 016163) and none of the fold-changes exceeded 1.9 or fell below −1.9, respectively. A higher fold-change due to an additional data set might be possible. However, based on our experience, a strong deviation is not expected. A similar result was obtained for the process control where only for one gene a significant alteration in the expression level of mRNA was found. Therefore, the materials used during the procedure of Soxhlet extraction as well as the used solvent DMSO *per se* had no major impact on changes in the abundance of transcripts at the highest applied concentration comparable to 30 mg SEQ/ml (DMSO concentration of 0.15%).

### Changes in the abundance of transcripts after whole sediment and extract treatments

**Figure 3:**
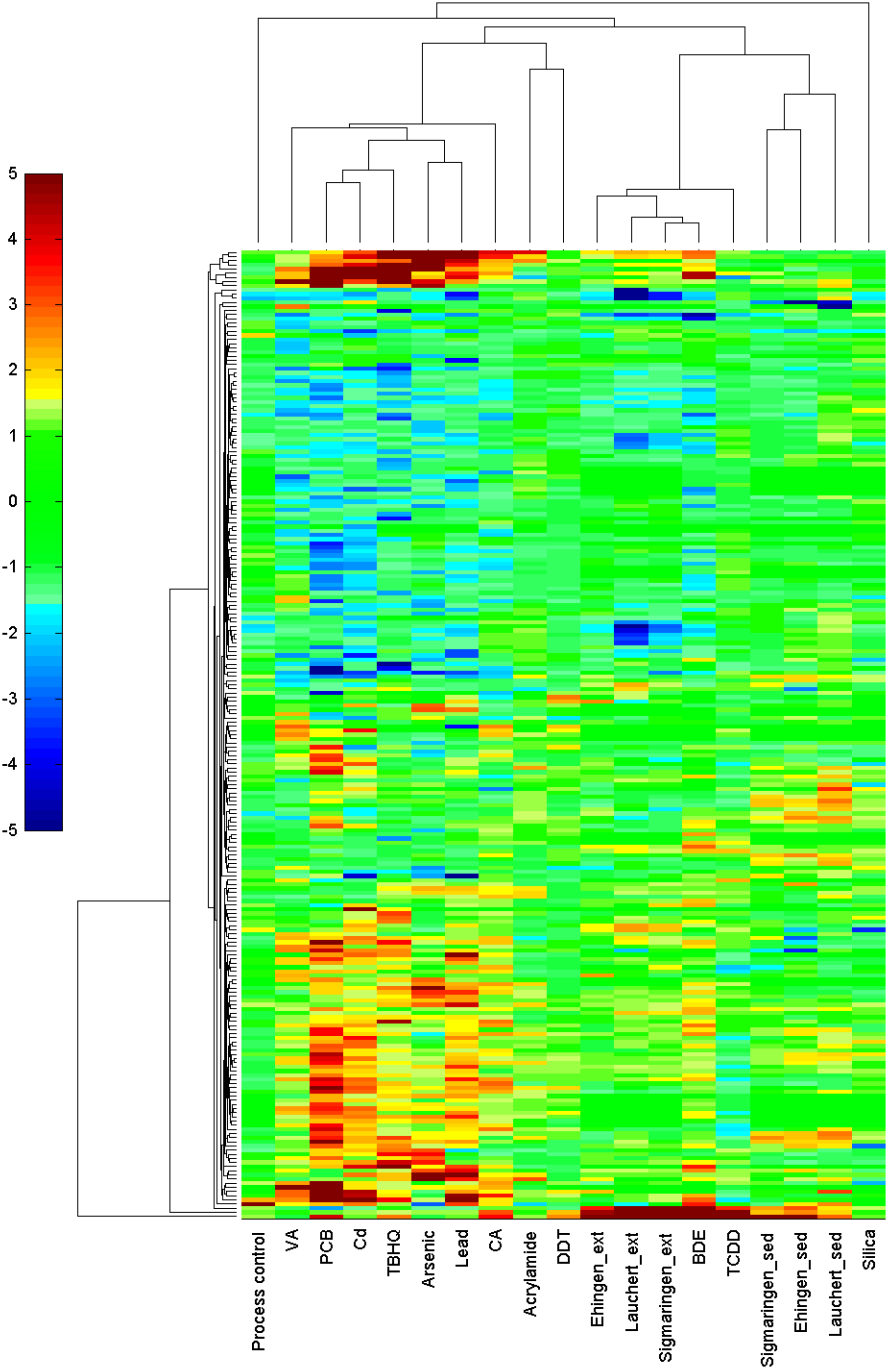
Hierarchical agglomerative cluster analysis of expression patterns of *Danio rerio* embryos tothree whole sediments and sediment extracts and 11 substances. (Yang et al. 2007; Acrylamide: 71 mg/l; Arsenic: arsenic (III) oxide, 79 mg/l; BDE: 2,2′4,4′-tetrabromo-diphenyl ether solution 47, 38.9 mg/l; CA: 4-chloroaniline, 50 mg/l; Cd: cadmium chloride, 5 mg/l; DDT: 1,1-bis-(4-chlorphenyl)-2,2,2-trichlorethane 15 mg/l; Lead: lead (II) chloride, 2.8 mg/l; PCB: Aroclor 1254, 33 mg/l; TBHQ: tert-butylhydroquinone, 1.7 mg/l; TCDD: 2,3,7,8-tetrachlorodibenzo-*p*-dioxin, 500 ng/l; VA: valproic acid, 50 mg/l). The cells are coloured according to the fold-change of a gene under a certain treatment. Blue segments represent genes with a strong decrease in the abundance of the corresponding transcript, red segments represent genes with a strong increase in the abundance of the corresponding transcript and green segments denote unaffected genes.

**Figure 4:**
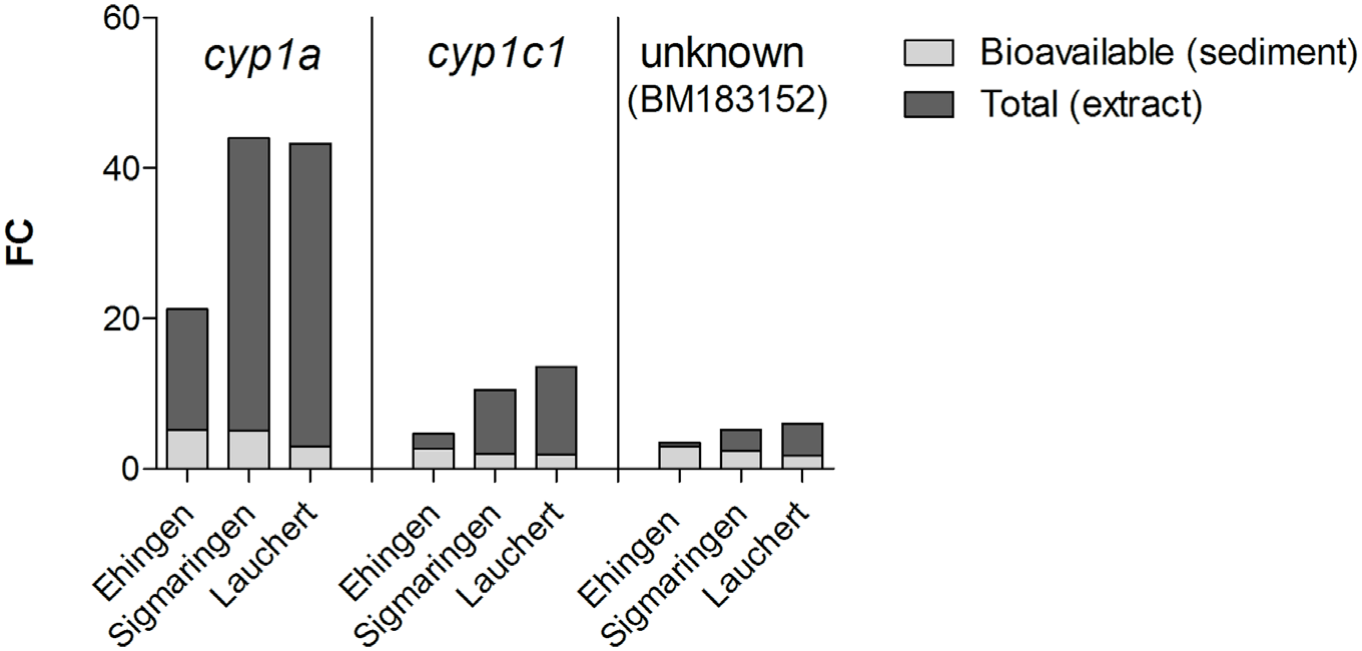
Changes in gene expression of *cyp1a*, *cyp1c1* and the unknown gene (BM183152) given in fold-changes (FC) for whole sediment and extract exposure scenarios to illustrate the contribution of bioavailable contaminants (light-grey bars) to the gene regulation induced by total organically extractable contaminants (dark-grey bars).

All analyses (significant alterations in the expression level of mRNAs, Table S1; cluster analysis, Figure 2; and profile of gene expression levels, Figure 3) revealed stronger correlations for whole sediment treatments among one another as well as sediment extract treatments among one another than for whole sediments and their corresponding extracts. This observation could be explained by differences in the availability of chemicals during whole sediment exposures compared to extract exposures. Sediment contact assays are more realistic approaches with high ecological relevance, but they only allow assessment of the bioavailable fraction of sediment-associated compounds due to the adsorption capacity of whole sediments. In contrast, testing of extracts represents an exposure to both easily available and strongly absorbed compounds that are organically extractable. Therefore, not only the amount of a chemical, but also the mixture of available chemicals differs between whole sediments and sediment extracts even of the same sample. Both the concentration of a chemical and the composition of chemicals in a sample can have an impact on alterations in the expression level of mRNAs. Yang et al. [3] showed that for most experiments the number of genes with an alteration in the expression level of mRNA increases and changes in the abundance of transcripts are greater if zebrafish embryos were exposed to higher concentrations of a chemical, e.g. *cyp1a* after exposure to TCDD. Thus, it is expected that a difference in the concentration of available chemicals lead to a discrepancy in the abundance of transcripts between whole sediment and sediment extract treatments of samples of the same location. Such a discrepancy is visible when comparing the abundance of transcripts in both whole sediment and extract treatments. Effect ratios between bioavailable and organically extractable pollutants in sediment samples are used to compare a toxic effect by naturally available compounds to the total toxic potential induced by the corresponding organic extracts as, e.g., presented for a genotoxic potential by Kosmehl et al. [17]. Such an approach can also be used to determine the contribution of bioavailable toxicants to the total alteration in gene expression levels of mRNA induced by the organic extracts. Therefore, fold-changes of the three exemplarily selected genes *cyp1a*, *cyp1c1* and an unknown gene (BM183152) are illustrated in Figure 4. Even though only one concentration was investigated for each sample and results would differ when testing higher or lower concentrations, the available data clearly show that sediment extract treatments, considered as worst-case exposure scenarios, induced higher fold-changes than the whole sediment exposure that only takes into account the bioavailable fraction of the sediment sample (Figure 4). Thus, greater alterations in gene expression levels of mRNA were observed for organically extractable compounds. Only the Ehingen whole sediment (bioavailable compounds) induced increased alterations in gene expression levels of mRNA of *cyp1c1* and BM183152 (>50%) in comparison to the sediment extract (total organically extractable compounds).

### Changes in the abundance of transcripts and the potential associations to results of biotests and chemical analyses

Among all genes with significant alterations in the expression level of mRNA, several are known to be regulated as a response to organic pollutants, for example *cyp1a and gst*.

#### Cytochrome P450 1A and glutathione S-transferase

The detection of CYP1A induction by means of EROD is a biomarker for the dioxin-like activity of environmental samples [29], [30] and has widely been used as a biomarker of environmental exposure of vertebrates to AhR agonists [31]. Chemical analysis and two biotests for Ah-receptor mediated toxicity (EROD assay with RTL-W1 cells and DR-CALUX with H4IIE cells) were performed with the sediment extracts used in this study [20]. The results documented a severe pollution of the sediments with AhR-inducing contaminants like PAHs, PCBs, polychlorinated dibenzodioxins and dibenzofurans (PCDDs and PCDFs) or further unknown substances. In addition, Otte et al. [32] examined EROD induction in gill filaments and liver of sticklebacks *in vivo* after exposure to the Sigmaringen sediment extract and found significant effects in both organs. These findings reveal a distinct contamination with AhR-inducing substances. In a comparison of different European river systems, sediments from the Danube River exhibited an Ah-receptor mediated toxicity as high as sediments from Rhine and Neckar [33]. Likewise, using the microarray assay in this study, a strong increase in the abundance of transcripts of *cyp1a* could be shown. It was apparently the highest regulated gene after extract exposures and among the highest regulated genes with regard to whole sediment exposures. Thus, the results are in accordance with the chemical analysis as well as results from previous studies and highlight the particular relevance of dioxin-like activity for the sediment samples of the Danube River. However, the ranking of the sediments regarding their AhR-inducing potential (cf. results of EROD- and DR-CALUX-assay, Table 1) were not in accordance with the strength of alterations in the expression level of mRNA of *cyp1a* at least for the sediment extract exposures (cf. Table S1). Among whole sediment exposures the Ehingen sample, although applied at a lower concentration (37.5 mg/ml) compared to the other samples (300 mg/ml), exhibited an equal *cyp1a* gene regulation (Table S1). Even if it is not possible to extrapolate the results for higher concentrations, the results indicated a greater impact of Ehingen sediment on alterations in the expression level of mRNA. Thus, with regard to whole sediments, a relation between the intensity of alterations in the expression level of mRNA and the toxic potential in biotests is assumed (cf. Table 1). However, this was not found for the investigation of sediment extracts. A stronger alteration in the expression level of mRNA of *cyp1a* was found for the Lauchert compared to the Ehingen sediment extract. This could be explained by the higher concentration of Lauchert sediment extract applied (30 mg SEQ/ml). However, a stronger change in the abundance of the transcripts was also induced by the Sigmaringen extract even though the Sigmaringen and Ehingen extracts were applied in the same concentration (10 mg SEQ/ml). Differences in the ranking of results from biotesting and analyses of alterations in the expression level of mRNA could be explained by several factors. (1) Different test species as well as whole embryos in comparison to cell cultures were used (EROD-assay with rainbow trout liver cells [20], DR-CALUX with rat hepatoma cells [20] and microarray experiments with zebrafish embryos in this study). Gene expression is species-, strain- and tissue-specific [34], [35], [36], [37], [38], [39] and especially in TCDD-responsiveness, species-specific differences are common [36], [37]. Furthermore, several tetracyclic PAHs activate the AhR pathway tissue-specifically inducing distinct patterns of *cyp1a* expression [39]. (2) The level of mRNA is not directly associated with the activity or the amount of the corresponding protein. A positive correlation between mRNA and protein expression levels can sometimes be possible as shown in a study with human monocytes [40] but an increased level of mRNA does not necessarily result in a higher synthesis rate or activity of the corresponding protein. The activity of proteins, for example, can be directly affected by chemical substances [41] and many important mechanisms for regulating cell functions are based on efficiency of translation and changes in protein stability or modification (i.e. post-translational and post-translational events [42], [43]). Those regulations are not detectable with profiles of gene expression levels. Therefore, an extrapolation from the strength of alteration in the expression level of mRNA to the results of biotests is hampered.

**Table 1:** Ecotoxicological potentials of the three investigated sediment samples.

Endpoint	Test system	Cell line/organism		Exposurescenario		Results	
					Lauchert	Sigmaringen	Ehingen
Cytotoxicity	Neutral red assay^[22]^	RTL-W1		extract	173 mgSEQ/ml (NR_50_)	36 mgSEQ/ml (NR_50_)	20 mgSEQ/ml (NR_50_)
Genotoxicity	Micronucleus test^[21]^	RTL-W1		extract	0.1 µg/g(NEQ)	0.9 µg/g(NEQ)	1.4 µg/g(NEQ)
	Comet assay^[19]^	RTL-W1		extract	CDI (x10) = 6.6	CDI (x10) = 16.4	CDI (x10) = 27.5
		*Danio rerio*		native	CDI (x100) = 1.6	CDI (x100) = 3.3	CDI (x100) = 40.8
Dioxin-like activity	EROD assay^[20]^	RTL-W1		extract	756 pg/g(BioTEQ)	2344 pg/g(BioTEQ)	2553 pg/g(BioTEQ)
	DR-CALUX assay^[20]^	H4IIE		extract	633 pg/g(BioTEQ)	1006 pg/g(BioTEQ)	2281 pg/g(BioTEQ)
Mutagenicity	Ames test^[22]^	*Salmonella* *typhimurium*	TA98+S9	extract	250 mgSEQ/ml(LOEC)	31.3 mgSEQ/ml(LOEC)	62.5 mgSEQ/ml(LOEC)
			TA98–S9	extract	500 mgSEQ/ml(LOEC)	n.e.	n.e.

CDI = Concentration-dependent induction factor.

NEQ = Nitroquinolin-*N*-oxid equivalents.

n.e. = no effect.

Regarding *gst*, different chemicals or sediment matrix, e.g. humic substances [44] can lead to an increase in the abundance of transcripts of *gst*, but a clear relation between a chemical and the gene regulation found in our study was not possible.

#### Natural Killer Cell Enhancing Factor

Another significant alteration was found for the abundance of the transcripts encoding the Natural Killer Cell Enhancing Factor (NKEF). The abundance of these transcripts was 2-fold greater in zebrafish embryos exposed to extracts. NKEF acts as an antioxidant [45] by increasing cellular resistance to oxidative damage by hydrogen peroxide and by protecting cells from alkyl hydroperoxide and metals such as methyl mercury [46]. Therefore, it can be used as a biomarker of oxidative stress or metal response. That implies that oxidative damage was potentially induced after exposure to Lauchert extract and Sigmaringen extract. However, an association to specific components of the extract is not possible, because oxidative stress can be caused by several substances and was even reported during AhR activation [47].

### Sets of genes encoding on different protein classes

Changes in the abundance of several transcripts characteristic for embryos exposed to whole sediments or sediment extracts were highlighted in different gene sets in Table S1. Gene set A and C comprise genes that were characteristic only for whole sediment or sediment extract exposures and did not appear for the other treatment. Gene set B includes genes with similar alterations in the expression level of mRNA after whole sediment and sediment extract exposures. These genes encoding on proteins related to xenobiotic metabolism were already discussed above and will be further discussed in chapter 4.4.

Set A represents genes encoding proteins mainly associated with digestion or fatty acid metabolism and with hydrolase activity. Several of these genes showed changes in the abundance of transcripts also after extract treatment (river Rhine sediment extracts) in the study by Kosmehl et al. [9], e.g., *ela2*, *ela2l*, *ela3l*, *ctsd*, *cpa4*, *cpa5*, *try, cel.1*. These responses might, therefore, be associated with the exposure to extracts. This is supported by the fact that the abundance of some of these transcripts were also found to be altered after exposure to the process control. Only the abundance of transcripts of one gene, *ela2l,* was significantly altered, but in consideration of the added fold-changes and tendencies depicted in the cluster transcript abundance of more genes in the gene set A (e.g. *ela2* and *ela3l*) tend to be similarly altered (Figure S1 and Table S1). However, the fold-changes were higher after sediment extract treatments with the exception of the Ehingen sediment extract. In addition, the changes in the abundance of transcripts were more distinct when the exposure lasted one day longer and extracts were tested at higher burden [9] compared to this study (Figure S1). Therefore, besides the influence of the extraction method as indicated by the process control, the time of exposure and the extracted substances might contribute to these gene regulations. Nevertheless, the profiles of gene expression levels of sediment extracts from different rivers indicate a rather similar tendency in gene regulation (Figure S1) even though both river Rhine sediments were stronger polluted especially with metals than the sediments from the Danube River. Further investigations are recommended but the results emphasize an impact of the exposure scenario on gene regulations.

A second set of genes (Set C) contained some genes encoding transcription factors. These genes, e.g. the *B-cell translocation gene 2* and the *Jun dimerization protein 2* were mainly regulated as a response to whole sediment exposure. There is no apparent explanation for the increase in the abundance of transcripts of these genes in the current study. Due to a lack of information about the target genes of the DNA binding proteins, interpretation of this regulation is yet impossible.

The comparatively low correlation of significantly regulated genes determined between the whole sediment treatments and their associated extract treatments revealed the high impact of a particular exposure scenario on the observed gene regulation.

### Comparison to patterns of the abundance of transcripts after exposure to single substances

Former investigations aimed at determining specific patterns of the abundance of transcripts of ecotoxicological relevant chemicals exposed to zebrafish embryos [3]. Based upon this, patterns of the abundance of transcripts resulting from whole sediment and sediment extract exposures were compared to the patterns of Aroclor 1254 (PCB) and ten single substances (Figure 3). One of the substances, BDE, will not be further discussed due to a contamination with furans which affected the regulation of genes, e.g., *cyp1a* [48]. The results show that a direct comparison between profiles of gene expression levels of single substances and those of whole sediments or sediment extracts is difficult, predominantly due to variations resulting from differences in time of exposure, length of exposure and concentrations applied. Patterns of the abundance of transcripts of single substances based upon an exposure lasting from 96 hpf to 120 hpf [3]. The zebrafish embryos were, therefore, one day older when RNA was extracted than the embryos exposed to whole sediments or sediment extracts in this study. It is not known how these differences in settings of experiments affect the results but an impact cannot be ruled out. Furthermore, Yang et al. [3] investigated, e.g., PCB at high concentrations where more than 250 genes were regulated. The PCB expression level profile showed low similarities to the profiles of whole sediments and sediment extracts. On the contrary, TCDD showed similarities in the abundance of transcripts compared to the sediment extracts, even though both TCDD and PCBs together with other dioxin-like substances were detected in the sediments by chemical analysis [20]. TCDD, whole sediments und sediment extracts were used at concentrations that caused low gene regulation. Therefore, even if all genes regulated for a whole sediment or extract would be similarly regulated for PCB, the correlation of treatments would be very low because most of the genes regulated for PCB were not regulated for one of the whole sediment or extract exposures. Consequently, whole sediment and sediment extract exposures correlated strong among each other and only slightly with PCB. To receive more information about the impact of concentrations of mixtures it would be interesting to see how profiles of gene expression levels would change after exposure to different concentrations of the same whole sediment or sediment extract. It would also be useful to apply single substances in lower concentrations and let the exposure last over a comparable period of time. This would result in a broader data basis and better comparable results.

In addition to differences in time of exposure and concentrations applied, additive, synergistic as well as potential antagonistic effects might impede a direct comparison. Already a mixture of only four metals causes recognizable changes in the profile of gene expression levels compared to single substances [3]. Sediments consist of a mixture of possibly several hundred substances. Profiles of gene expression levels of these complex mixtures will hardly correlate with profiles of single substances and therefore, contaminants can hardly be detected based on a comparison of expression level profiles.

Beside the detection of stressor-specific signatures, microarray experiments were already used for the identification of biomarkers. For example, former studies showed the possibility to identify biomarkers for drug-activity [49], for an improved diagnosis of ulcerative colitis patients [50] and for the infection of salmon with *Piscirickettsia salmonis* [51]. In these cases, the genes encoding information on the newly identified biomarkers were already identified. However, in this study, a number of candidate genes for further analysis as potential novel biomarkers of exposure to whole sediments or sediment extracts were highlighted. BM 183152 is a candidate for in-depth investigation, because changes in the abundance of transcripts of this gene were revealed for most of the sediment exposures in our study and it was found to be regulated as cellular response to benzo[*a*]pyrene exposure by Lam et al. [52], but the function remains unknown.

## Conclusion and Outlook

The investigations with oligonucleotid microarrays elucidated changes in the abundance of transcripts in zebrafish embryos exposed to whole sediments or sediment extracts. General differences in the abundance of transcripts between whole sediment and sediment extract treatments were found. According to multivariate analysis, the impact on the abundance of transcripts is less influenced by the sample (site of sampling) than by the method of exposure (whole sediment/extract). This should be considered for future analyses of the abundance of transcripts when investigating exposures to sediment samples. Moreover, the impact could be directly related to the exposure scenarios that represent either the bioavailable or the extractable fraction, because an influence of silica dust (to dilute the whole sediments) and DMSO (solvent, contained in the extracts) on the abundance of transcripts was only marginal and would not explain the differences found for the exposure scenarios. Overall, the extractable fraction (sediment extract) had a greater impact on the abundance of transcripts than the bioavailable fraction (whole sediment).

For both exposure scenarios the abundance of the transcript encoding the well-known biomarker CYP 1A could be detected. The increase in the abundance of transcripts of *cyp1a* was in concordance with the results from two Ah-receptor-mediated bioassays and the analysis of dioxin-like contaminants (e.g. PAHs, PCBs, PCDDs/Fs). However, a comparability of the strength of gene regulation and results of biotests is not given. Additional specific correlations between the abundance of transcripts and biological effects were not observed, but the results of the microarray approach indicate an exposure to unspecific stress-inducing compounds, which might be explained by the use of whole organisms, and consequently, the lack of detection of specific gene responses occurring in particular tissues.

Finally, the data obtained clearly documented the impact of exposure scenarios on the abundance of transcripts and the need for further studies to provide a broader data basis and a better understanding to interpret results.

## Supporting Information

Figure S1 **Profiles of gene expression levels of extracts from Danube River sediments compared to sediment extracts from the river Rhine.** The profile of a process control is also presented. The highlighted set of genes includes down-regulated genes encoding proteins associated with digestion or fatty acid metabolism and hydrolase activity. Several of these genes were similarly regulated after treatment with extracts regardless of the sampling locations.
(PDF)

Table S1 Significant alterations in the expression level of mRNAs with a fold-change ≥2 and an adjusted p-value≤0.025 for whole sediment and sediment extract treatments. Fold changes are listed for the corresponding gene names. A (green background) marks genes encoding proteins mainly with hydrolase activity. B (purple background) marks genes encoding enzymes related to xenobiotic metabolism. C (red background) marks genes encoding proteins related to DNA damage response or to DNA/mRNA binding. Investigated were whole sediments and sediment extracts from the sampling sites Sigmaringen (Sig), Ehingen (Ehi) as well as the tributary Lauchert (Lau) along the Danube River in Germany.
(PDF)
