# Supplementary material for: Impacts of Different Exposure Scenarios on Transcript Abundances in Danio rerio Embryos when Investigating the Toxicological Burden of Riverine Sediments
Source: PLoS One. 2014 Sep 4;9(9):e106523. doi: 10.1371/journal.pone.0106523 (PMC4154683; doi:10.1371/journal.pone.0106523)
Supplement: Figure S1 — Profiles of gene expression levels of extracts from Danube River sediments compared to sediment extracts from the river Rhine. The profile of a process control is also presented. The highlighted set of genes includes down-regulated genes encoding proteins associated with digestion or fatty acid metabolism and hydrolase activity. Several of these genes were similarly regulated after treatment with extracts regardless of the sampling locations. (PDF) [file pone.0106523.s001.pdf]

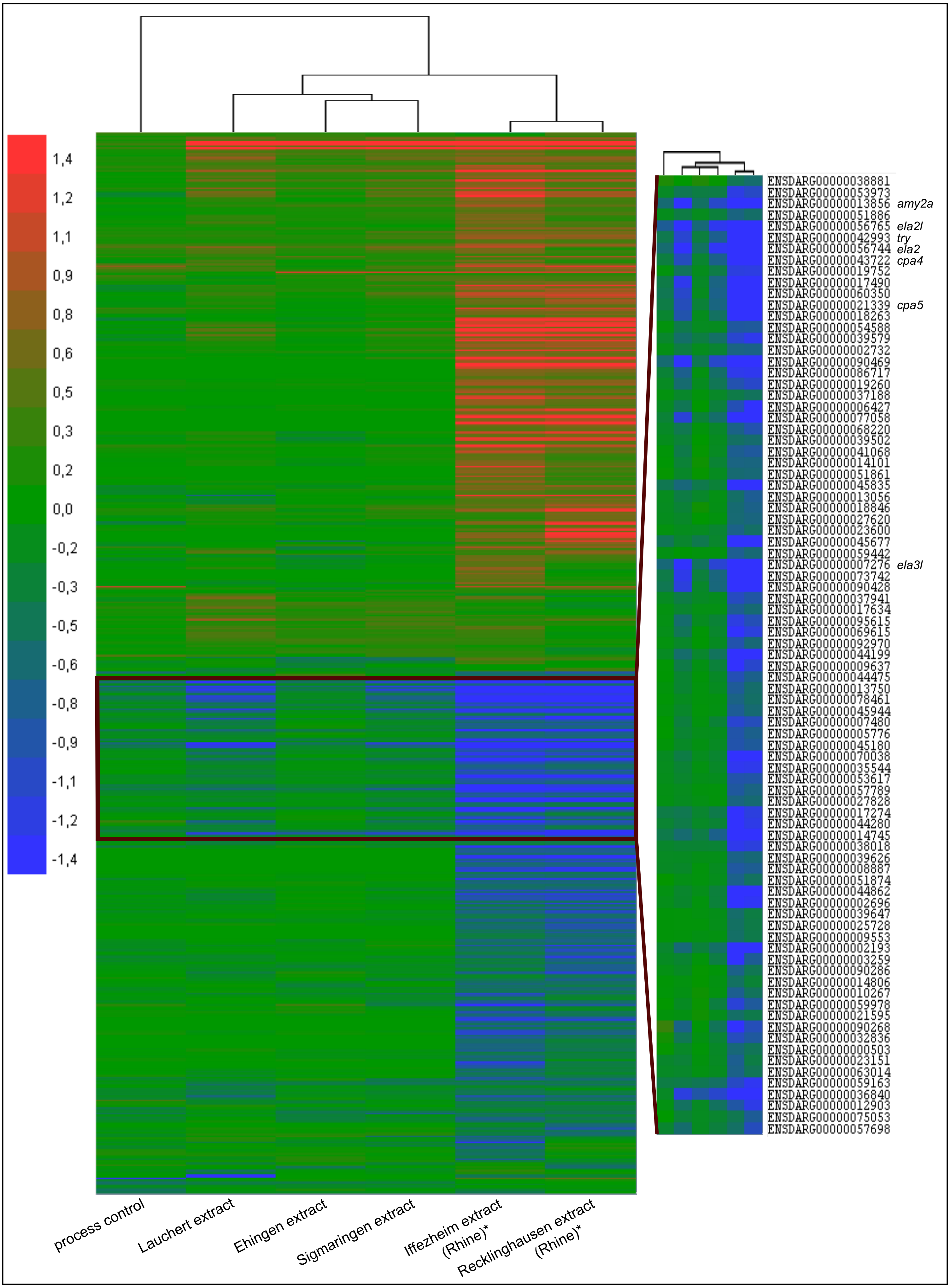

Figure S1. Profiles of gene expression levels of extracts from Danube River sediments compared to sediment extracts from the river Rhine. The profile of a process control is also presented. The highlighted set of genes includes down-regulated genes encoding proteins associated with digestion or fatty acid metabolism and hydrolase activity. Several of these genes were similarly regulated after treatment with extracts regardless of the sampling location.

* Data according to Kosmehl et al. [9] (reanalysed)
