# Supplementary material for: Impacts of Different Exposure Scenarios on Transcript Abundances in Danio rerio Embryos when Investigating the Toxicological Burden of Riverine Sediments
Source: PLoS One. 2014 Sep 4;9(9):e106523. doi: 10.1371/journal.pone.0106523 (PMC4154683; doi:10.1371/journal.pone.0106523)
Supplement: Table S1 — Significant alterations in the expression level of mRNAs with a fold-change ≥2 and an adjusted p-value≤0.025 for whole sediment and sediment extract treatments. Fold changes are listed for the corresponding gene names. A (green background) marks genes encoding proteins mainly with hydrolase activity. B (purple background) marks genes encoding enzymes related to xenobiotic metabolism. C (red background) marks genes encoding proteins related to DNA damage response or to DNA/mRNA binding. Investigated were whole sediments and sediment extracts from the sampling sites Sigmaringen (Sig), Ehingen (Ehi) as well as the tributary Lauchert (Lau) along the Danube River in Germany. (PDF) [file pone.0106523.s002.pdf]

Table S1 Significant alterations in the expression level of mRNAs with a fold-change ≥ 2 and an adjusted p-value ≤ 0.025 for whole sediment and sediment extract treatments. Fold changes are listed for the corresponding gene names. A (green background) marks genes encoding proteins mainly with hydrolase activity. B (purple background) marks genes encoding enzymes related to xenobiotic metabolism. C (red background) marks genes encoding proteins related to DNA damage response or to DNA/mRNA binding. Investigated were whole sediments and sediment extracts from the sampling sites Sigmaringen (Sig), Ehingen (Ehi) as well as the tributary Lauchert (Lau) along the Danube River in Germany.

		**Sediment**				**Extract**				
		**Lau sediment**	**Sig sediment**	**Ehi sediment**	**Silica dust**	**Lau extract**	**Sig extract**	**Ehi extract**	**Process control**	
**Name or orthologous (ensembl genname)**	**GenBank ID**	300 mg/ml	300 mg/ml	37.5 mg/ml	600 mg/ml	30 mg SEQ/ml	10 mg SEQ/ml	10 mg SEQ/ml	30 mg SEQ/ml	
**Antioxidant**										
Similar to natural killer cell enhancing factor (zgc:110343)	BI980610	-	-	-	-	2.3	2.0	1.7[b]	-	
**Hydrolase activity**										
Amylase, alpha 2A; pancreatic (*amy2a*)	BM103972	-	-	-	-	-4.2	-2.9	-1.7[b]	-1.8[b]	
Similar to elastase 1 precursor (B8JM76_DANRE)	BM154197	-	-	-	-	-3.2	-	-	-	
Elastase 2 (*ela2*)	BI705588	-	-	-	-	-7.9	-3.7	-1.7[b]	-	
Elastase 2 like (*ela2l*)	AW174168	1.8[b]	-	-	-	-7.6	-3.6	-1.9[b]	-2.2	
Elastase 3 like (*ela3l*)	AI558632	-	-	-	-	-4.9	-3.1	-1.7[b]	-1.9[b]	**A**
Carboxypeptidase A4 (*cpa4*)	AI496860	-	-	-	-	-2.8	-2.1	-	-	
Carboxypeptidase A5 (*cpa5*)	AF376130		-	-	-	-2.7	-2.0	-	-	
Cathepsin K (*ctsk*)	AI384696	-	-	2.4	-	-	-	-	-	
Cationic trypsin-3 (*try*)	AJ297822	-	-	-	-	-3.0	-2.1	-	-	
Similar to chymotrypsinogen 2	BM101561	-	-	-	-	-3.2	-	-	-	
Similar to trypsinogen	BM184009	-	-	-	-	-3.5	-2.0	-	-	
**Lipid metabolism**										
Carboxyl ester lipase (*cel.1*)	AF003943	-	-	-	-	-3.6	-2.0	-	-	
Similar to apolipoprotein D (LOC100148022)	BM101644	-	-	-	-	-3.0	-2.1	-	-	
**Oxidoreductase activity**										
Cytochrome P450 1A (*cyp1a*)	AF057713	3.0	5.1	5.2	-	43.3	44.0	21.2	-	**B**
CytochromeP4501C1(*cyp1c1*)	BG738243	1.9[c]	2.0	2.7[a]	-	13.6	10.5	4.7	-	
Glutathione S-transferase pi	AF285098	-	-	-	-	2.4	2.2	2.2	-	
Similar to sulfotransferase	AI959735	-	-	-	-	-	-	3.8	-	
**Involved in cytokinesis**										
Protein regulator of cytokinesis 1 (zgc:55664)	BG304231	-	-	-	-	2.3	-	-	-	
Suppressor of cytokine signaling 3 (*socs3a*)	BI878700	2.5	2.6	2.1	-	-	-	-	-	
**Regulator of innate immunity**										
Dual specificity phosphatase 1 (*dusp1*)	BI888604	2.1	-	-	-	-	-	-	-	
**Structural molecule**										
Similar to keratin	BE200701	-	-	-	-	-2.1	-	-	-	
Keratin 15 (*krt15*)	AI397347	-	-	-	-	-3.3	-3.0	-2.4	-	
Type I cytokeratin	AF197880	-	-	-	-	-2.0	-	-	-	

Name or orthologous (ensamble genname)	GenBank ID	Whole sediment samples: Lau sediment 300 mg/ml	Sig sediment 300 mg/ml	Ehi sediment 37.5 mg/ml	Silica dust 600 mg/ml	Extract samples: Lau extract 30 mg SEQ/ml	Sig extract 10 mg SEQ/ml	Ehi extract 10 mg SEQ/ml	Process control 30 mg SEQ/ml
**DNA damage response**									
B-cell translocation gene 2 (*btg2*)	AB036784	2,8	2.1[a]	-	-	-	-	-	-
**DNA/mRNA binding**									
mRNA-decapping enzyme 1A (*dcp1a*)	AJ344448	2.1	-	-	-	-	-	-	-
TSC22 domain family protein 3 (*tsc22d3*)	AW018476	2.2	-	-	-	-	-	-	-
Distal-less homeobox protein 3b (*dlx3b*)	X65060	2.2	2.1[a]	-	-	-	-	-	-
CCAAT/enhancer binding protein beta (*cebpb*)	AW019436	2.0	2.1	2.0	-	-	-	-	-
Similar to CCCH zinc finger protein (hypothetical protein LOC564559; zgc:162730)	BM184163	2.1	-	-	-	-	-	-	-
Jun dimerization protein 2	BI326453	4,7	4.0	4.4[a]	-	-	-	-	-
Kruppel-like factor 2a (*klf2a*)	AF392992	-	2.0	2.0	-	-	-	-	-
Kruppel-like factor 2b (*klf2b*)	AF392995	-	2.1	2.6	-	-	-	-	-
Similar to nuclear receptor (*nr0b2*)	BI892036	-	-	2.0	-	-	-	-	-
Nuclear receptor subfamily 1 group D member 1 (*nr1d1*)	BM024131	2.1	-	2.2	-	-	-	-	-1.8[b]
Transcription factor (*sp9*)	BI979064	-	-	-	-	2.1	-	-	-
Similar to Transcription factor CP2-like 4 (si:dkey-221l4.7)	AI477969	-	-	-	-	2.4[a]	2.1	-	-
**Miscellaneous**									
Annexin A1b (*anxa1b*)	AI331515	-4.3	-2.6	-5.3	-	-	-	-	-
Cation transport regulator-like protein 1 (*chac 1*)	BI888548	2.1	-	-	-	-	-	-	-
Prostaglandin-endoperoxide synthase 2a (*ptgs2a*)	AW077995	2.7	2.0[a]	2.0[a]	-	-	-	-	-
Protein disulfide isomerase family A, member 2 (*pdia 2*)	AW154400	-	-	-	-	-2.5	-	-	-
Interleukin 11 receptor, alpha (*il11ra*)	AW154333	2.3	-	-	-	-	-	-	-
Coenzyme Q-binding protein COQ10 homolog (zgc:73324)	BI880133	2.2	-	-	-	-	-	-	-
Secreted class III semaphorin (*sema3d*)	AF124485	2.2	-	-	-	-	-	-	-
Similar to estrogen-responsive B box protein (LOC767638, zgc: 153258)	BI878269	-	-	-	-	2.4	2.1	-	-
Nucleoside phosphorylase (*np*)	AW019173	2.5	-	2.5	-	-	-	-	-
Unknown	AA566708	-	-	-	-	-6.9	-3.0	-	-
Unknown	AF112374	2.3 [a]	2.1	-	-	-	-	-	-
Unknown	AI353541	-2.6	-2.9	-3.0	-	-	-	-	-
Unknown	AI353694	2.0	2.4	-	-	2.2	1.8[b]	-	-
Unknown	AI437134	2.0	-	-	-	-2.1[a]	-	-	-
Unknown	AL591482	-	-	-	-	2.4	-	-	-
Unknown	AW455027	-	-	-2.8	-	-	-	-	-
Unknown (wu:fk66d05)	BE016163	2.0	-	-	-	-	-	-	-
Unknown	BE557704	2.8	2.7	3.1	-	-	-	-	-
Unknown	BG737292	-	-	-	-	-2.1	-2.0	-	-
Unknown	BI878214	-	-	-5.3	-	2.2[a]	-	-	
Unknown	BI474700	-	-	-	-	2.6	-	-	-

C

		Whole sediment samples				Extract samples			
		**Lau sediment**	**Sig sediment**	**Ehi sediment**	**Silica dust**	**Lau extract**	**Sig extract**	**Ehi extract**	**Process control**
**Name or orthologous (ensamble genname)**	**GenBank ID**	300 mg/ml	300 mg/ml	37.5 mg/ml	600 mg/ml	30 mg SEQ/ml	10 mg SEQ/ml	10 mg SEQ/ml	30 mg SEQ/ml
Unknown (LOC556307; zgc:158605)	BM095918	-	-	2.0	-	-	-	-	-
Unknown	BM101698	-	-	-	-	2.6	-	-	-
Unknown LOC568780 (zgc:158343)	BM102743	2.0	-	-	-	-	-	-	-
Unknown (LOC100148860)	BM183152	1.8[b]	2.4	3.0	-	6.0	5.2	3.5	-
Unknown	BQ618331	-	-	-	-	-	2.0	-	-

[a] added fold changes if fold change is |≥ 2.0| and the adjusted p-value ≤ 0.05

[b] added fold changes if fold change is |≤ 2.0| but |≥ 1.7| and the adjusted p-value ≤ 0.005

[c] added fold change with a fold change |≥ 2.0| and an adjusted p-value of 0.028
